# Unveiling the hidden risk: paternal smoking and alcohol exposure prior to conception as independent factors for allergic rhinitis in children

**DOI:** 10.3389/fped.2024.1394400

**Published:** 2024-05-30

**Authors:** Junrong Chen, Xiaohua Liu, Wenwen Su, Zixin Liu, Guoying Sun, Yide Yang, Mei Tian, Jian Li, Yunpeng Dong

**Affiliations:** ^1^ Key Laboratory of Model Animals and Stem Cell Biology in Hunan Province, School of Medicine, Hunan Normal University, Hunan, China; ^2^ Nursing Department, Changde Vocational Technical College, Changde, Hunan, China; ^3^ Department of Medical Consortium Work, Changsha Hospital for Maternal & Child Health Care Affiliated to Hunan Normal University, Changsha, Hunan, China; ^4^ Hunan Provincial People's Hospital Affiliated to Hunan Normal University, Changsha, Hunan, China; ^5^ Third Xiangya Hospital, Central South University, Changsha, Hunan, China; ^6^ Key Laboratory of Study and Discovery of Small Targeted Molecules of Hunan Province, School of Medicine, Hunan Normal University, Changsha, Hunan, China; ^7^ The Engineering Research Center of Reproduction and Translational Medicine of Hunan Province, Changsha, China; ^8^ Department of Otolaryngology-Head and Neck Surgery, Yichang Central People's Hospital, Three Gorges University, Hubei, China

**Keywords:** paternal smoking before pregnancy, paternal alcohol consumption before pregnancy, children allergic rhinitis, DOHaD, disease susceptibility

## Abstract

**Introduction:**

Limited knowledge exists regarding the impact of paternal smoking and alcohol exposure on the development of allergic rhinitis in offspring. Our study aimed to investigate the potential association between preconception paternal smoking and alcohol exposure and the likelihood of children allergic rhinitis.

**Methods:**

A retrospective case-control study of 556 prepubertal children aged 3–12 years was performed. The participants were 278 children with allergic rhinitis and 278 healthy controls matched for age and gender. Self-administered questionnaires were distributed and collected on-site, focusing on various factors related to the children's fathers, mothers, and the children themselves during the first year of life and the past 12 months, from March to October 2022.

**Results:**

Multivariate analysis demonstrated that paternal smoking, paternal alcohol consumption prior to conception, paternal allergic diseases, children with a family history of allergies, maternal allergic diseases and pregnancy complications were identified as independent risk factors for allergic rhinitis in their offspring. Moreover, after considering confounding factors, it was observed that paternal smoking exceeding 5 cigarettes per day in the year preceding pregnancy and exceeding 11 years significantly elevated the likelihood of allergic rhinitis in children (OR = 2.009 and 2.479, respectively). Furthermore, the consumption of alcohol by the father at intervals of less than one month in the year prior to pregnancy and a duration of alcohol consumption exceeding 11 years prior to pregnancy are both associated with a significantly increased risk of allergic rhinitis in children (OR = 2.005 and 3.149, respectively).

**Conclusions:**

Paternal smoking and alcohol consumption prior to conception contribute to an increased risk of allergic rhinitis in children, with the risk being dependent on the dosage and duration of exposure. Therefore, it is important to not only focus on personal and maternal environmental exposures when considering the occurrence risk of allergic rhinitis in children, but also to consider paternal detrimental exposures prior to conception.

## 1 Introduction

Allergic rhinitis, a non-infectious chronic inflammatory condition affecting the nasal mucosa, is attributed to exposure to specific allergens. Common symptoms include sneezing, runny nose, nasal irritation, and nasal congestion, with potential ocular manifestations (1). Global studies indicate an approximate worldwide incidence rate of 12.66% in children (2). Notably, the prevalence of allergic rhinitis in China has risen from 15.79% in 2016 (3) to 22.00% (4) in 2021. Allergic rhinitis (AR) is poorly controlled. Children afflicted with allergic rhinitis may experience nasal irritation, fatigue, disrupted sleep, cognitive impairment, reduced memory capacity, diminished learning efficiency, and ongoing medical costs, which can significantly lower their quality of life and that of their family (5).

The prevalence of allergic rhinitis in children is primarily influenced by genetic and environmental factors. However, these factors alone do not fully account for the high incidence of allergic rhinitis. In 1989, Barker firstly published research findings indicating that elderly individuals with a history of low birth weight or malnutrition during infancy were at a heightened risk for cardiovascular disease. In 1990, he introduced the "fetal origin of adult diseases (FOAD) hypothesis," (6). Which later evolved into the "Developmental Origin of Health and Diseases (DOHaD)" theory after extensive researches. According to this view, adverse exposures during early life stages, including sperm and oocyte development, zygote formation, fetal development, infancy, and childhood, can have long-term effects on the health and susceptibility of offspring to illness (7).

Previous research primarily focused on mothers and found that maternal factors such as age between 26 and 35 years old (8), exposure to air pollution (9) during pregnancy, passive smoking (10) and gestational hypertension (11) can elevate the likelihood of allergic rhinitis in their offspring. However, recent studies have increasingly highlighted the significant contribution of fathers in shaping the health and disease susceptibility of their children.

A multitude of detrimental factors, such as cigarette smoking and excessive alcohol consumption, have been found to have a negative impact on sperm quality (12). Pre-conception alcohol intake by fathers has been associated with an elevated likelihood of behavioral problems (13) and microcephaly in their offspring (14). Furthermore, research has indicated that paternal smoking prior to pregnancy may influence the development of asthma in their children (15). However, little is known about the effects of paternal smoking and alcohol exposure on the offspring's allergic rhinitis until now. Hence, this study will utilize the DOHaD theory as a foundation to examine the association between pre-conception factors in fathers and the prevalence of allergic rhinitis in their progeny. Special consideration will be devoted to exploring the potential influence of modifiable factors, such as paternal smoking and alcohol exposure, on the susceptibility of children to developing allergic rhinitis.

## 2 Methods

### 2.1 Study design and population

**Figure 1:**
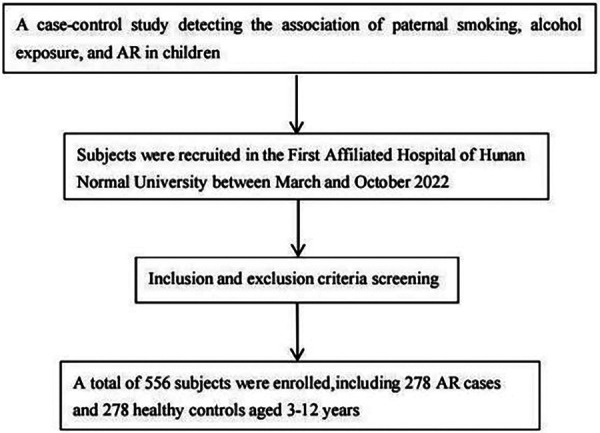
Flowchart of recruitment of subjects in a case-control study.

This retrospective case-control study was conducted at the First Affiliated Hospital of Hunan Normal University between March and October 2022. A total of 278 who were treated for allergic rhinitis and their parents were invited to participate in the case group, while 278 children who received physical examinations in the hospital and their parents were included in the control group. The study received approval from the Ethics Committee of the Hunan Normal University (approval number: 2022277), and informed written consent was obtained from all participants. All procedures for data collection follow-strict quality assurance and quality control guideline. Strict inclusion and exclusion criteria were applied for patient recruitment.The screening flowchart of the subjects is shown in Figure 1.

#### 2.1.1 Inclusion criteria for case group

(1) Children who have been diagnosed with allergic rhinitis based on the diagnostic criteria of allergic rhinitis. (2) Children who are single births. (3) Children aged between 3 and 12 years old. (4) The child's father does not have any communication barriers and has no history of mental illness.

#### 2.1.2 Exclusion criteria for case group

(1) Children who have previously suffered from immune diseases or severe organic diseases, as well as children with mental illnesses. (2) The child's father is uncooperative and does not have sufficient time to receive a questionnaire survey.

#### 2.1.3 Inclusion criteria for control group

(1) Children have no allergic rhinitis. (2) Children who are single births. (3) Children aged between 3 and 12 years old. (4) The child's father does not have any communication barriers and has no history of mental illness.

#### 2.1.4 Exclusion criteria for control group

(1) Children who have previously suffered from immune diseases or severe organic diseases, as well as children with mental illnesses. (2) The child's father is uncooperative and does not have sufficient time to receive a questionnaire survey.

### 2.2 Diagnostic criteria for allergic rhinitis in children

According to the Chinese guidelines for the Diagnosis and Treatment of Allergic Rhinitis in Children (1), these criteria require a comprehensive assessment of the allergy history, clinical symptoms, and laboratory examination results. ① Allergy history: the occurrence of allergy conditions and allergies in close relatives, including mothers and/or fathers. ② Typical clinical symptoms include sneezing, clear nasal discharge, itching, and nasal congestion. These symptoms may also be accompanied by respiratory symptoms like coughing and eye symptoms like itching and weeping. ③ Physical symptoms: The main sign of AR attack is bilateral nasal mucosal swelling pale, swollen inferior turbinate, with excessive amount of clear water like secretion in the nasal cavity. ④ Laboratory testing: a skin prick test (SPT) to identify at least one positive allergen. Detecting the presence of serum-specific IgE antibodies has high specificity and sensitivity. Additionally, the examination of nasal secretions revealed the presence of eosinophils exceeding 0.05%, and a regular blood test indicated an increase in eosinophil count.

### 2.3 Clinical data collection of the participants

(1) Factors related to the newborn within a year of birth: birth weight, delivery model, gravidity and parity, gestational age, mode of conception, allergic diseases, feeding methods within four months, family history of allergies, place of residence, time of adding complementary foods, types of complementary food, living environment (such as house decoration, dampness, purchase of new furniture, long-term use of blankets and carpets, frequent exposure to stuffed toys, daily ventilation time, and presence of pets), smoking habits of family members, frequency of colds, and antibiotic usage.
(2) Factors related to the child within 12 months: age, height, body weight, allergy history, place of residence, living environment (such as house decoration, dampness, purchase of new furniture, long-term use of blankets and carpets, frequent exposure to stuffed toys, daily ventilation time, presence of pets, and regular classroom ventilation), frequent utilization of air conditioning, children's lifestyle and habits [such as insufficient daily sleep duration (<8 h), daily vegetable consumption, and daily fruit consumption], as well as smoking habits of family members.
(3) Factors related to the mother: mother's age before pregnancy, height and weight before pregnancy, weight gain during pregnancy, pregnancy complications, allergic diseases, history of urticaria, and passive smoking during pregnancy.
(4) Factors related to the father: age before pregnancy, height and weight before pregnancy, birth weight, gestational age, educational level, income, engaging in high-risk occupations before pregnancy, allergic diseases, history of urticaria, pre-pregnancy hypertension, pre-pregnancy diabetes, and smoking and alcohol consumption in the year before pregnancy.

### 2.4 Statistical analysis

Continuous variables with normally distributed data are expressed as means and standard deviations, and a *t*-test was used to compare variables between two groups. The median and quartiles (Q1–Q3) were utilized for non-normally distributed continuous variables, and the Mann-Whitney non-parametric test was used to compare the variables between groups. Cases (*n*) and percentages (%) were employed to represent categorical variables, while the chi-square test was utilized to evaluate the qualitative data. The case–control study was matched according to gender frequency, and the baseline data of age and nationality were compared by chi-square test or nonparametric rank sum test. Include statistically significant variables from univariate analysis into multivariate analysis, and multivariate logistic regression (Forward Wald method) was used to calculate the odds ratio (OR) and 95% confidence interval (CI) to evaluate the relationship between various factors and childhood allergic rhinitis. A *P* value less than 0.05 was considered statistically signiﬁcant. Data were analyzed using SPSS for Windows version 26.

## 3 Results

### 3.1 Participant characteristics

In total, 556 children with complete data were included (57.9% boys, *n* = 322; 42.1% girls, *n* = 234), with the majority of children being Han Chinese (95.1%). According to the most recent data from the previous 12 months, it was discovered that more kids in the case group have a history of allergies than in the control group (83.8% vs. 9.0%, *p *< 0.01). However, the study groups were relatively homogeneous in terms of nationality, age, body weight, and height. Detailed data are shown in Table 1.

**Table 1:** Characteristics of included cases and controls.

Variables		Case group (%)	Control group (%)	*χ*^2^/*Z*	*P*
Nationality	Han	264 (95.0)	265 (95.3)	0.390	0.844
	Minority	14 (5.0)	13 (4.7)		
Age (year)		6.1 (5.0–8.4)	6.0 (4.8–7.9)	−0.876	0.381
Gender	Boy	161 (57.9)	161 (57.9)		
	Girl	117 (42.1)	117 (42.1)		
Height (cm)		120.0 (109.8, 130.0)	118.0 (107.0, 129.3)	−1.610	0.107
Weight (kg)		21.0 (17.0, 28.0)	20.0 (17.0, 26.0)	−1.184	0.237
Allergy history^a^	Yes	233 (83.8)	25 (9.0)	312.871	<0.01

^a^ Including drug, milk, peanut, flour, egg, seafood, dust mite, pollen.

### 3.2 Univariate analysis

#### 3.2.1 Children indicators

Statistically significant differences were observed between the case group and control group in various factors. These factors include family allergy history (37.1% vs. 11.9%, *P *< 0.01), exclusive breastfeeding during the first four months of life (67.6% vs. 75.7%, *P* = 0.039), urban residency (82.0% vs. 73.3%, *P* = 0.019), purchasing new home furniture (14.4% vs. 8.6%, *P* = 0.033), and experiencing frequent colds during the first year of life (22.3% vs. 14.7%, *P* = 0.022). Furthermore, within the past 12-month period, notable disparities were observed between the two groups concerning variables such as urban residency (91.0% vs. 85.3%, *P* = 0.036), familial smoking habits (56.8% vs. 46.8%, *P* = 0.017), and frequent use of air conditioning (38.5% vs. 26.3%, *P* = 0.002). These data are shown in Tables 2–5.

**Table 2:** Characteristics regarding basic information at birth.

Variables		Case group (%)	Control group (%)	*χ* ^2^	*P*
Parity	First born	195 (70.1)	182 (65.5)	1.392	0.238
	Non-first born	83 (29.9)	96 (34.5)		
Method of conception	Natural pregnancy	270 (97.1)	269 (96.8)	0.061	0.805
	ART	8 (2.9)	9 (3.2)		
Birth weight	<2,500 g	14 (5.0)	14 (5.0)	1.364	0.506
	2,500–4,000 g	260 (93.5)	256 (92.1)		
	≥4,000 g	4 (1.4)	8 (2.9)		
Mode of delivery	Spontaneous birth	173 (62.2)	171 (61.5)	0.222	0.895
	Caesarean section without labor	62 (22.3)	60 (21.6)		
	Caesarean section with Labor	43 (15.5)	47 (16.9)		
Gestational age	Preterm	19 (6.8)	19 (6.8)	1.032	0.597
	Full-term	253 (91.0)	249 (89.6)		
	Post-term	6 (2.2)	10 (3.6)		

ART, assisted reproductive technology.

**Table 3:** Characteristics regarding allergies and feeding history within first year of birth.

Variables		Case group (%)	Control group (%)	*χ* ^2^	*P*
History of allergic diseases^a^	Yes	76 (27.3)	25 (9.0)	31.469	<0.01
	No	202 (72.7)	253 (91.0)		
Allergy history^b^	Yes	67 (24.1)	35 (12.6)	12.295	<0.01
	No	211 (75.9)	243 (87.4)		
Family allergy history	Yes	103 (37.1)	33 (11.9)	47.696	<0.01
	No	175 (62.9)	245 (88.1)		
Feeding methods within four months	Exclusive breastfeeding	188 (67.6)	210 (75.5)	4.279	0.039
	Non-exclusive breastfeeding	90 (32.4)	68 (24.5)		
Time of adding complementary foods	Within 4 months of birth	15 (5.4)	23 (8.3)	1.808	0.179
	After 4 months of birth	263 (94.6)	255 (91.7)		
Types of complementary food	1–2 types	108 (38.8)	118 (42.4)	0.746	0.388
	3–5 types	170 (61.2)	160 (57.6)		

^a^ Including wheezing and eczema.

^b^ Including drug, milk, peanut, flour, egg, seafood, dust mite, pollen.

**Table 4:** Characteristics regarding the living environment of children within first year after birth.

Variables		Case group (%)	Control group (%)	*χ* ^2^	*P*
Place of residence	Urban only	228 (82.0)	205 (73.7)	5.523	0.019
	Not just cities	50 (18.0)	73 (26.3)		
House decoration	Yes	14 (5.0)	16 (5.8)	0.141	0.707
	No	264 (95.0)	262 (94.2)		
Damp	Yes	36 (12.9)	33 (11.9)	0.149	0.700
	No	242 (87.1)	245 (88.1)		
Purchasing new furniture	Yes	40 (14.4)	24 (8.6)	4.520	0.033
	No	238 (85.6)	254 (91.4)		
Long term use of blankets and carpets	Yes	9 (3.2)	14 (5.0)	1.134	0.287
	No	269 (96.8)	264 (95.0)		
Frequently exposed to Stuffed toy	Yes	59 (21.2)	46 (16.5)	1.984	0.159
	No	219 (78.8)	232 (83.5)		
Daily ventilation time	<8 h	33 (11.9)	43 (15.5)	1.543	0.462
	8–12 h	111 (39.9)	105 (37.8)		
	>12 h	134 (48.2)	130 (46.8)		
Keeping pets	Yes	12 (4.3)	16 (5.8)	0.602	0.438
	No	266 (95.7)	262 (94.2)		
Family members smoking	Yes	157 (56.5)	150 (54.0)	0.356	0.551
	No	121 (43.5)	128 (46.0)		
Frequent colds	Yes	62 (22.3)	41 (14.7)	5.255	0.022
	No	216 (77.7)	237 (85.3)		
Antibiotic use	Never	147 (52.9)	154 (55.4)	4.850	0.088
	≤3 times	102 (77.9)	109 (39.2)		
	>3 times	29 (22.1)	15 (5.4)		

**Table 5:** Characteristics regarding the living environment of children in the past 12 months.

Variables		Case group (%)	Control group (%)	*χ* ^2^	*P*
Place of residence	Cities only	253 (91.0)	237 (85.3)	4.401	0.036
	Not just cities	25 (9.0)	41 (14.7)		
House decoration	Yes	14 (5.0)	17 (6.1)	0.307	0.579
	No	264 (95.0)	261 (93.9)		
Damp	Yes	31 (11.2)	28 (11.9)	0.171	0.680
	No	247 (88.8)	250 (88.1)		
Purchase new furniture	Yes	23 (8.3)	24 (8.6)	0.023	0.879
	No	255 (91.7)	254 (91.4)		
Long term use of blankets and carpets	Yes	13 (4.7)	8 (2.9)	1.237	0.266
	No	265 (95.3)	270 (97.1)		
Frequently exposed to Stuffed toy	Yes	47 (16.9)	44 (15.8)	0.188	0.731
	No	231 (83.1)	234 (84.2)		
Daily ventilation time	<8 h	28 (10.1)	18 (6.5)	3.125	0.210
	8–12 h	125 (45.0)	120 (43.2)		
	>12 h	125 (45.0)	140 (50.4)		
Keeping pets	Yes	15 (5.4)	22 (7.9)	1.419	0.234
	No	263 (94.6)	256 (92.1)		
Classroom is regularly ventilated	Yes	264 (97.8)	271 (98.2)	0.117	0.733
	No	6 (2.2)	5 (1.8)		
Family members smoking	Yes	158 (56.8)	130 (46.8)	5.648	0.017
	No	120 (43.2)	148 (53.2)		
Frequent use of air conditioning	Yes	107 (38.5)	73 (26.3)	9.497	0.002
	No	171 (61.5)	205 (73.7)		
Daily sleeping<8 h^a^	Yes	1 (0.4)	4 (1.4)	0.807	0.369
	No	277 (99.6)	274 (98.6)		
Daily consumption of vegetables	Never	4 (1.4)	6 (2.2)	1.301	0.522
	Occasionally	102 (36.7)	112 (40.3)		
	At least once per day	172 (61.9)	160 (57.6)		
Daily consumption of fruits^a^	Never	0 (0.0)	1 (0.4)	3.293	0.186
	Occasionally	109 (39.2)	93 (33.5)		
	At least once per day	169 (60.8)	184 (66.2)		

^a^ Continuity-adjusted Chi-square test.

#### 3.2.2 Maternal related indicators

Significant differences were observed between the two groups in terms of maternal allergic diseases (15.5% vs. 2.9%, *P *< 0.01), pre-pregnancy weight (Q1∼Q3: 45.0∼53.0 vs. 47.0∼55.0, *P *< 0.01), and pregnancy complications (14.0% vs. 5.8%, *P* = 0.001). Additional details can be found in Table 6.

**Table 6:** Characteristics regarding mother's related factors.

Variables		Case group (%)	Control group (%)	*Z/χ* ^2^	*P*
Age of conception (years)		27.0 (25.0, 30.0)	27.0 (25.0, 30.0)	−1.028	0.304
Pre-pregnancy height (cm)		160.0 (157.8, 162.0)	160.0 (158.0, 163.0)	0.150	0.881
Pre-pregnancy weight kg)		50.0 (45.0, 53.0)	50.0 (47.0, 55.0)	−3.685	<0.01
Pre-pregnancy BMI (kg/m^2^)^a^	<18.5	92 (33.1)	69 (24.8)	9.199	0.034
	18.5–23.9	175 (62.9)	185 (66.5)		
	24–27.9	9 (3.2)	22 (7.9)		
	≥28	2 (0.70)	2 (0.70)		
Weight gain during pregnancy		12.0 (9.0, 16.0)	12.0 (10.0, 16.0)	−0.217	0.828
History of allergic diseases	Yes	43 (15.5)	8 (2.9)	26.445	<0.01
	No	235 (84.5)	270 (97.1)		
History of urticaria	YES	8 (2.9)	9 (3.2)	0.061	0.805
	No	270 (97.1)	269 (96.8)		
Pregnancy complications^b^	Yes	39 (14.0)	16 (5.8)	10.674	0.001
	No	239 (86.0)	262 (94.2)		
Passive smoking during pregnancy	Yes	79 (28.4)	73 (26.3)	0.326	0.568
	No	199 (71.6)	205 (73.7)		

^a^ Continuity-adjusted Chi-square test.

^b^ Pregnancy complications include: pregnancy hypertension, pregnancy diabetes, pregnancy anemia, pregnancy biliary syndrome, pregnancy heart disease, pregnancy viral hepatitis; BMI, body mass index.

#### 3.2.3 Paternal related indicators

The study revealed significant associations between various paternal indicators and the outcomes of interest. Specifically, there were higher rates of paternal allergic disease (35.3% vs. 7.6%, *P *< 0.01), paternal pre-pregnancy hypertension (4.3% vs. 1.4%, *P* = 0.042), paternal smoking in the year before pregnancy (61.2% vs. 48.6%, *P* = 0.021), and paternal alcohol consumption in the year before pregnancy (51.1% vs. 37.8%, *P* = 0.002) in the case group. For more comprehensive information, please refer to Table 7.

**Table 7:** Characteristics regarding paternal related factors.

Variables		Case group (%)	Control group (%)	*Z/χ* ^2^	*P*
Age of conception (years)		29.0 (26.0, 32.0)	29.0 (26.0, 33.0)	−0.296	0.767
Pre-pregnancy height (cm)		172.0 (169.0, 175.0)	172.0 (169.0, 175.0)	−0.060	0.952
Pre-pregnancy weight kg)		68.0 (61.8, 75.0)	70.0 (62.8, 75.0)	−0.537	0.591
Pre-pregnancy BMI (kg/m^2^)	<18.5	14 (5.0)	14 (5.0)	0.754	0.860
	18.5–23.9	167 (60.1)	163 (58.6)		
	24–27.9	85 (30.6)	92 (33.1)		
	≥28	12 (4.30)	9 (3.2)		
Birth weight	Low birth weight;	13 (4.7)	8 (2.9)	1.570	0.456
	Normal birth weight	257 (92.4)	264 (95.0)		
	High birth weight	8 (2.9)	6 (2.2)		
Gestational age^a^	Preterm	12 (4.3)	6 (2.2)	2.574	0.276
	Full-term	263 (94.6)	267 (96.0)		
	Post-term	3 (1.1)	5 (1.8)		
Education level	Junior middle school	20 (7.2)	24 (8.6)	0.494	0.920
	Senior high school	61 (21.9)	63 (22.7)		
	Junior college	89 (32.0)	87 (31.3)		
	Bachelor degree or above	108 (38.8)	104 (37.4)		
Average monthly income before pregnancy (RMB)^a^	<3,000	5 (1.8)	4 (1.4)	0.973	0.808
	3,000–5,000	71 (25.5)	63 (22.7)		
	5,000–8,000	101 (36.30)	101 (36.3)		
	>8,000	101 (36.3)	110 (39.6)		
Engaging in high-risk occupations before pregnancy^b^	Yes	29 (10.4)	24 (8.6)	0.521	0.470
	No	249 (89.6)	254 (91.4)		
History of allergic diseases	Yes	98 (35.3)	21 (7.6)	63.391	<0.01
	No	180 (64.7)	257 (92.4)		
History of hives	Yes	4 (1.4)	5 (1.8)	0.000	1.000
	No	274 (98.6)	273 (98.2)		
Pre-pregnancy hypertension	Yes	12 (4.3)	4 (1.4)	4.119	0.042
	No	266 (95.7)	274 (98.6)		
Pre-pregnancy diabetes^a^	Yes	5 (1.8)	3 (1.1)	0.127	0.722
	No	273 (98.2)	275 (98.9)		
Smoking in the year before pregnancy	Yes	170 (61.2)	135 (48.6)	5.328	0.021
	No	108 (38.8)	143 (51.4)		
Drinking alcohol in the year before pregnancy	Yes	142 (51.1)	105 (37.8)	9.973	0.002
	No	136 (48.9)	173 (62.2)		

^a^ Continuity-adjusted Chi-square test; BMI, body mass index.

^b^ Yes: Engaged in professions such as chemical engineering, hair dressing, bread and cake making, art teacher, wood processing, interior decoration, rubber and plastic manufacturing, leather and fur processing.

### 3.3 Multivariate analysis

**Figure 2:**
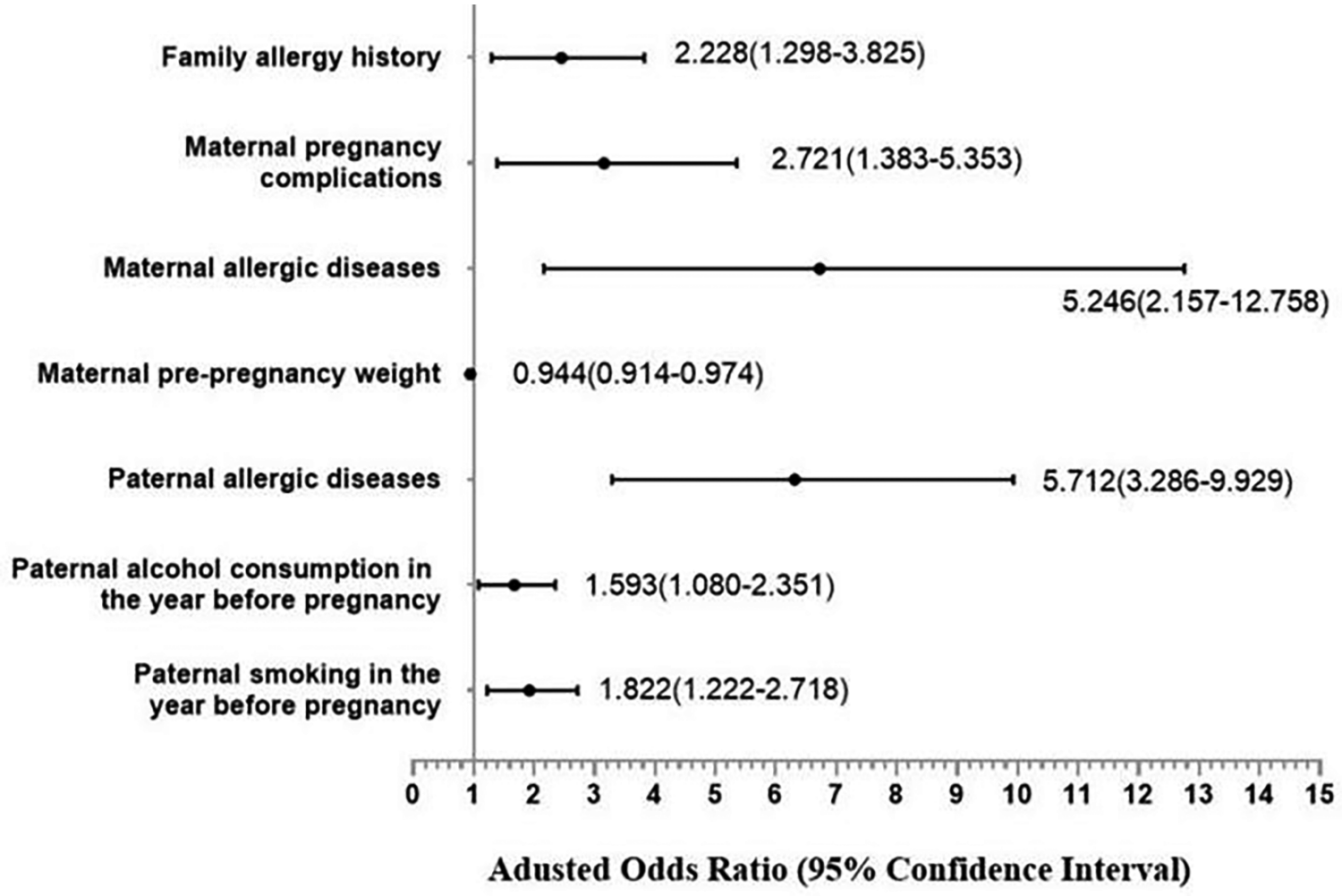
Forest plot of multivariate analysis.

Based on the findings of the univariate analysis, it was observed that a total of 18 covariates exhibited statistically significant differences between the case and control groups. Subsequently, through the integration of professional expertise, the categories of “children with allergic diseases within one year of age” and “children with allergic history within the past 12 months” were excluded from the multivariate regression analysis. The results of the multivariate analysis indicate that several independent risk factors contribute to the development of allergic rhinitis in offspring. These factors include a family history of allergies (OR = 2.228, 95% CI: 1.298–3.825), maternal allergic diseases (OR = 5.246, 95% CI: 2.157–12.758), pregnancy complications (OR = 2.721, 95% CI: 1.383–5.353), paternal allergic diseases (OR = 5.712, 95% CI: 3.286–9.929), paternal smoking in the year before pregnancy (OR = 1.822, 95% CI: 1.222–2.718), and paternal alcohol consumption in the year before pregnancy (OR = 1.593, 95% CI: 1.080–2.351). Shown in Table 8 and Figure 2.

**Table 8:** Multivariate analysis results.

Variables	*β*	S.E	Wald	OR	95% CI	*P*
Family allergy history	0.801	0.276	8.448	2.228	1.298–3.825	0.004
Maternal pre-pregnancy weight	−0.058	0.016	12.603	0.944	0.914–0.974	<0.01
Maternal allergic diseases	1.657	0.453	13.362	5.246	2.157–12.758	<0.01
Maternal pregnancy complications	1.001	0.345	8.406	2.721	1.383–5.353	0.004
Paternal allergic diseases	1.743	0.282	38.160	5.712	3.286–9.929	<0.01
Paternal smoking in the year before pregnancy	0.600	0.204	8.650	1.822	1.222–2.718	0.003
Paternal drinking alcohol in the year before pregnancy	0.466	0.199	5.500	1.593	1.080–2.351	0.019

### 3.4 Correlation analysis between paternal smoking and alcohol consumption before pregnancy and children's allergic rhinitis

**Figure 3:**
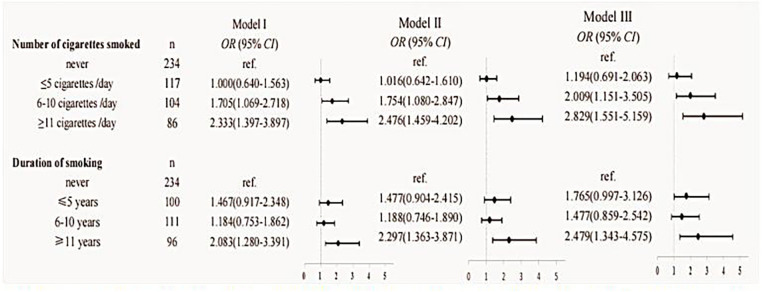
Results of the analyses assessing the association between paternal smoking and children's allergic rhinitis. Model I: unadjusted. Model II: adjusted for the age of conception, educational level, income, child age, child height and weight, and child's place of residence. Model III: adjusted for age of conception, educational level, income, child age, child height and weight, child's place of residence, family allergy history, paternal allergic disease, paternal alcohol consumption in the year before pregnancy, maternal weight before pregnancy, maternal allergic disease, and pregnancy complications.

The following analysis aimed to examine two key modifiable factors pertaining to fathers prior to conception, with a first focus on the quantity of cigarettes smoked per day in the year before pregnancy and the duration of smoking in relation to children's allergic rhinitis. Three distinct binary logistic regression models were adjusted to examine the association between paternal smoking habits and the risk of allergic rhinitis in offspring. Our findings indicate that a heightened risk of allergic rhinitis in children is observed when the father consumes more than 5 cigarettes per day in the year before pregnancy (OR = 2.341, 95% CI: 1.458–3.758). Furthermore, a statistically significant increase in the odds ratio was observed as the number of cigarettes smoked per day escalated from exceeding 5 cigarettes. Although the relationship between the duration of smoking years and the risk of allergic rhinitis in offspring is inconclusive, nevertheless, our research indicates a noteworthy elevation in the risk of allergic rhinitis among children when the father has engaged in smoking for a minimum of 11 years prior to conception (OR = 2.479, 95% CI: 1.551–5.159), as evidenced across all three models. Detailed data can be seen in Figure 3.

**Figure 4:**
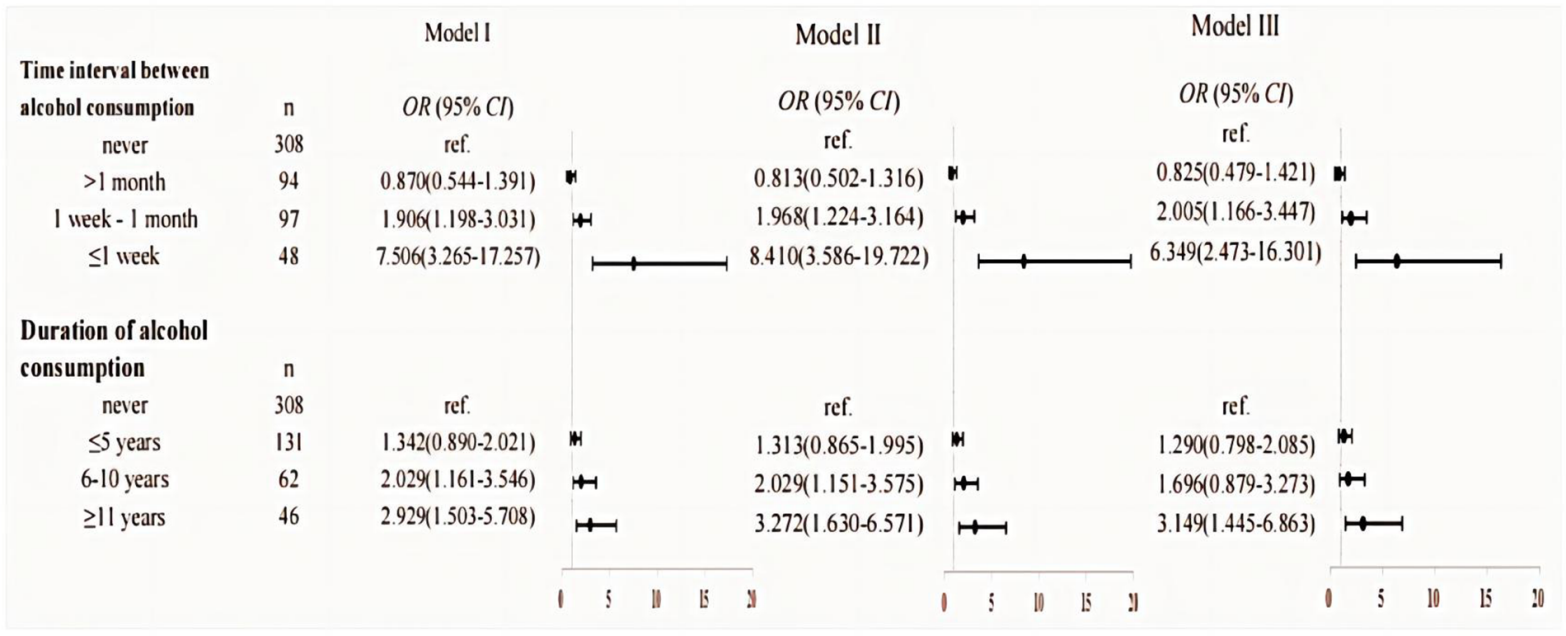
Results of the analyses assessing the association between paternal alcohol consumption and children's allergic rhinitis. Model I: unadjusted. Model II: adjusted for the age of conception, educational level, income, child age, child height and weight, and child's place of residence. Model III: adjusted for age of conception, educational level, income, child age, child height and weight, child's place of residence, family allergy history, paternal allergic disease, paternal smoking in the year before pregnancy, maternal weight before pregnancy, maternal allergic disease, and pregnancy complications.

And then we focus on the frequency and timing of alcohol consumption in relation to children's allergic rhinitis. The findings, supported by the upward trend observed in the overall OR value, indicate a significant correlation between the frequency or time of alcohol consumption and the occurrence of allergic rhinitis in their offspring. All three models demonstrated that paternal alcohol consumption less than one month before pregnancy and consuming alcohol for more than 11 years before conception may significantly elevate the risk of allergic rhinitis in offspring (OR = 2.668, 95% CI: 1.639–4.345 and OR = 3.149, 95% CI: 1.445–6.863, respectively). Detailed data can be seen in Figure 4.

In summary, the data indicates a dose-and-time correlation between the level or duration of preconception paternal smoking or alcohol consumption and the incidence of allergic rhinitis in offspring.

## 4 Discussion

The research reveals that fathers who engaged in smoking and alcohol consumption prior to conception significantly increased the risk of their offspring developing allergic rhinitis, with a positive correlation observed between the severity of paternal habits and the likelihood of their children being affected by the condition.

Prior studies have primarily examined the impact of maternal active smoking, maternal passive smoking during pregnancy, and child passive smoking on the development of allergy diseases in children. Many studies have indicated that maternal exposure to passive smoking during pregnancy is associated with various adverse outcomes, including gestational diabetes (16), low birth weight infants (17), small head circumference at birth (18), neural tube defects and mental health issues in offspring (19), and childhood allergic diseases, including wheezing, asthma, and allergic rhinitis, in offspring (20–22). Similarly, a recent meta-analysis has shown that children exposed to passive smoking are at an increased risk of developing allergic rhinitis (4). However, our study showed that the smoking of family members in the past 12 months were not significant risk factor in the multivariate analysis.

However, the neglect of the fact that fathers are the predominant smokers has been evident. According to the findings of the 2018 National Adult Tobacco Epidemic Survey, the prevalence of smoking among individuals aged ≥15 years in China stands at 26.6%. This rate is further disaggregated by gender, revealing that 50.5% of smokers are male, while only 2.1% are female (23). This study discovered paternal active smoking before pregnancy increased the development of allergy diseases in children. Our findings are consistent with prior research studies. Specifically, Lu et al. (24) have emphasized the association between paternal smoking and azoospermia, while Knudsen et al. (25) have identified a correlation between paternal smoking initiation before pregnancy and increased adult body mass index in their progeny. Furthermore, Kim et al. (26) have provided evidence indicating that paternal smoking both before and during pregnancy significantly heightens the risk of autism spectrum disorders in offspring. Moreover, evidence indicates that paternal prenatal smoking can result in epigenetic alterations that heighten the susceptibility of their progeny to asthma (15). A comprehensive investigation encompassing 24,168 parents of children unveiled a positive correlation between paternal smoking prior to conception and an elevated likelihood of asthma in their offspring, whereas maternal smoking prior to conception does not yield a similar impact (27).

Prior studies have shown that starting to smoke before puberty may result in a decline in lung function in offspring, especially when compared to dads who never smoke (28). Similar studies have revealed that fathers who start smoking after the age of 15 are linked to a higher BMI in their offspring (25). Intriguingly, a dose-effect relationship was found in the effect of paternal smoking on the risk of offspring allergic rhinitis. We found that paternal smoking more than 5 cigarettes per day in the year prior to pregnancy and more than 11 years increases the probability of offspring developing allergic rhinitis by 1.009 and 1.479 times, respectively. These are consistent with some previous studies. A cohort study carried out in the Netherlands found that smoking by the father for one month prior to conception was associated with a higher risk of offspring being small for gestational age, and the relative risk of smoking more than 10 cigarettes per day was higher than that of smoking less than 10 cigarettes per day (29). Conversely, other investigations have indicated that paternal smoking of at least one cigarette per day within the month prior to conception does not exhibit any significant association with either gestational age or birth weight of the progeny (30). It is presently believed that exposure to tobacco smoke may result in an immunological imbalance in Th1/Th2 cells, thereby serving as the underlying mechanism by which passive smoking in children heightens the likelihood of developing allergic diseases. Tobacco smoke can detrimentally impact children's lung development and exert cytotoxic, pro-inflammatory, and anti-inflammatory effects on nasal epithelial cells. Furthermore, a wealth of evidence suggests that oxidative stress acts as a mediator in allergy rhinitis (31, 32). According to Loffredo's research, children who are exposed to passive smoking have more Nox2 activity than the control group, which can result in oxidative stress reactions (33).

Moreover, the mechanism underlying the prenatal effects of tobacco smoke can be predominantly elucidated by alterations in epigenetic mechanisms, including histone acetylation, microRNA expression, and DNA methylation, in conjunction with individual genetic susceptibility (34). Therefore, further study is necessary to explore the exact mechanism by which allergic diseases are induced by paternal cigarette smoke.

Similarly to smoking, the majority of studies primarily examine the impact of alcohol abuse during pregnancy on fetal development and the health of subsequent generations. Prenatal alcohol abuse has been identified as a notable contributor to the increasing incidence of sudden neonatal death (35). Maternal alcohol consumption at moderate to high levels during pregnancy can result in physical and neurocognitive dysplasia in infants (36), and depression in their offspring (37). It is interesting to report a significant increase in the likelihood of atopic eczema before the ages of 3 and 5 due to maternal alcohol consumption during pregnancy (38). Similar studies have also revealed an elevated incidence of early-onset atopic dermatitis in children born to moms who consume four or more cups of alcohol per week at 30 weeks of pregnancy (39).

Just like the correlation observed with paternal smoking, scholarly investigations have also substantiated the susceptibility of offspring to emotional, physical, and socioeconomic adversities when their fathers engage in alcohol misuse. Experimental studies conducted on animals have revealed that the persistent exposure of fathers to alcohol prior to conception can impede the development of the placenta in their offspring fetuses (40). Research conducted in human clinical studies has also demonstrated that offspring of fathers who consume alcohol at least once a week during the three months preceding pregnancy face an elevated susceptibility to various adverse outcomes, including anxiety and depression, physical ailments, sleep disturbances, cognitive impairments in girls, and aberrant behaviors in boys (13). Noteworthy, this study showed that the risk of anxiety, depression, and sleep problems in 4-year-old girls increased with the father's weekly cumulative drinking dose in three months before pregnancy (13). According to Zuccolo et al.'s study (41), the likelihood of microcephaly in offspring at birth increased with the amount of alcohol the father consumed prior to becoming pregnant. While some research indicates that children born to mothers who drank alcohol at least once a month before pregnancy had greater birth weights than children born to moms who drank alcohol less than once a month, fathers were not associated in a similar way (30). Still knowledge gaps exist in the associations between paternal alcohol abuse and allergic diseases. Our findings made up for this gap by revealing that drinking alcohol one year before pregnancy was an independent risk factor for allergic rhinitis in children aged 3–12 years old. Furthermore, our findings indicate that there is a shorter interval between alcohol consumption and a higher risk of allergic rhinitis in offspring, especially when the interval between alcohol intakes is less than one week, there is a 5.349-fold increase in the risk of allergic rhinitis in offspring. Additionally, a longer duration of alcohol consumption is associated with a greater risk of allergic rhinitis in offspring. Specifically, paternal alcohol consumption exceeding 11 years prior to conception can raise the risk of allergic rhinitis in children by 2.149 times.

The consumption of alcohol by pregnant women poses a risk to the developing fetus, as it exposes them to the harmful effects of alcohol and its byproducts. Additionally, there is evidence suggesting that alcohol consumption during pregnancy may lead to an increase in IgE levels in the umbilical cord blood (42). In certain animal models investigating the association between paternal alcohol consumption and offspring, it has been observed that chronic alcohol intake negatively impacts sperm quality compared to a control group. Furthermore, RNA sequencing analysis has revealed notable differences in gene expression related to the testis between the alcohol-exposed group and the control group. These findings suggest that long-term alcohol exposure may disrupt the balance of the gut microbiota (43). Recently, the rat model, in which the paternal exposure to a combination of nicotine, ethanol, and caffeine demonstrated that such mixed adverse exposure resulted in heightened activity of the hypothalamic pituitary adrenal axis, diminished sperm quality, unfavorable outcomes in pregnancy, abnormal indicators of fetal serum metabolism, and dysfunction in multiple organs in male rats. Notably, the reduced activity of the glucocorticoid-insulin-like growth factor 1 (GC-IGF1) axis may serve as the primary mechanism underlying the developmental and multi-organ dysfunction observed in offspring due to paternal mixed exposure (44).

**Figure 5:**
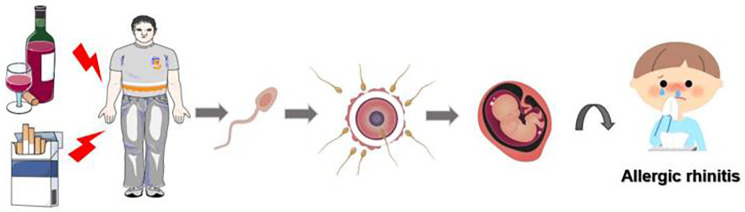
Schematic representation illustrating the potential impact of paternal smoking and alcohol exposure prior to conception on the likelihood of allergic rhinitis development in offspring.

In conclusion, our research findings demonstrate that paternal smoking and alcohol consumption prior to pregnancy are significant risk factors for the development of allergic rhinitis in children. The potential impact of paternal smoking and alcohol consumption on sperm function may have repercussions on embryonic development, potentially heightening the susceptibility of offspring to allergic rhinitis (Figure 5). As smoking and alcohol consumption are modifiable factors, our results highlight the importance of community hospitals and healthcare providers in raising awareness about men's pre-pregnancy health state, emphasizing the necessity for men to cease or reduce their alcohol and tobacco consumption. However, it is important to note that this study is limited in its retrospective nature and single-center clinic setting. Therefore, future investigations need to consider prospective, multicentric, and mechanistic studies to further explore this topic.

## Data Availability

The original contributions presented in the study are included in the article/Supplementary Material, further inquiries can be directed to the corresponding authors.
